# Circulating B cell and T cell activation states predict clinical outcomes in melanoma and reveal dynamic immune reinvigoration with checkpoint inhibitor immunotherapy

**DOI:** 10.1136/jitc-2026-015585

**Published:** 2026-08-07

**Authors:** Lucy Booth, Rebecca Adams, Angela Clifford, Francisco Aguilar, Nadira Ali, Cynthia Bishop, Jahangir Sufi, Yin Wu, Amanda Fitzpartick, Jenny L C Geh, Alastair D MacKenzie Ross, Hawys Lloyd-Hughes, Matthew Stodell, Claire S Daniel, Sean Whittaker, Khushboo Sinha, Zena N Willsmore, Manuela Terranova-Barberio, Niwa Ali, Katie E Lacy, Thomas J Tull, Sophia Tsoka, Sophia N Karagiannis

**Affiliations:** 1 St. John’s Institute of Dermatology, School of Basic and Medical Biosciences and KHP Centre for Translational Medicine, King’s College London, Guy's Hospital, London, UK; 2 Department of Informatics, Faculty of Natural, Mathematical and Engineering Sciences, King’s College London, Strand Campus, London, UK; 3 Advanced Cytometry Platform, R&D Department, Guy's and St Thomas’ NHS Foundation Trust, Guy's Hospital, London, UK; 4 Department of Medical Oncology, Guy's and St Thomas’ Hospitals NHS Foundation Trust, London, UK; 5 Breast Cancer Now Research Unit, School of Cancer and Pharmaceutical Sciences, King's College London, Innovation Hub, Guy's Cancer Centre, London, UK; 6 Centre for Inflammation Biology and Cancer Immunology, School of Immunology & Microbial Sciences, King's College London, London, UK; 7 Department of Plastic Surgery, Guy's and St Thomas’ NHS Foundation Trust, London, UK; 8 St John’s Institute of Dermatology, Guy's and St Thomas’ Hospitals NHS Foundation Trust, London, UK; 9 Head and Neck Cancer Department, Guy's and St Thomas’ Hospitals NHS Foundation Trust, London, UK; 10 Adnexal Department, Moorfields Eye Hospital NHS Foundation Trust, London, UK; 11 Barts Cancer Institute, Queen Mary University of London, London, UK; 12 Peter Gorer Department of Immunobiology, King’s College London, London, UK; 13 Centre for Gene Therapy and Regenerative Medicine, King’s College London, London, UK

**Keywords:** B cell, Immune Checkpoint Inhibitor, Skin Cancer, Adaptive, T cell

## Abstract

**Background:**

Nearly half of patients with melanoma do not respond to immune checkpoint inhibitors (CPIs) and many develop immune-related adverse events (irAEs), often forcing treatment discontinuation, and underscoring the need to predict and monitor outcomes. Responses may depend on both B cell and T cell activity.

**Methods:**

We performed high-dimensional mass cytometry profiling of coexisting peripheral B cell and key T cell states in treatment-naïve patients and healthy individuals, and paired longitudinal samples from CPI-treated patients, with clinical annotations to define immune correlates of outcomes.

**Results:**

CPI-naive patients exhibited reduced CD19+ B cells, reduced B cell (CD21, IL-2, CXCR5) and T cell (CD38, CD27) activation markers, alongside enriched naïve (CD21lo) and double-negative (DN2)-like B cells, CD95+IL-10+plasmablasts, consistent with extrafollicular responses. Concurrently, programmed cell death protein 1 (PD-1)+ and proliferation marker protein-67 (Ki67)+ T cell expansion indicates ongoing activation with features of proliferative exhaustion. Active disease featured increased regulatory CD95 expression on B cells and expanded T follicular helper-like and activated DN (CD4−CD8−) T cells, indicating sustained antigen stimulation. Pretreatment, elevated PD-1+ T cells predicted irAEs, whereas VEGF (vascular endothelial growth factor)+TGF-β (transforming growth factor-β)+ DN T cells were enriched in patients without subsequent toxicity. Pretreatment, plasmablasts, transitional B cells, Forkhead box protein P3 (FoxP3)+ and central memory-like CD8+ T cells correlated with worse overall survival; naïve CD21lo B cells, PD-1+CD8+ T cells and CD4+follicular helper-like T cells predicted shorter event-free survival; CD4+ memory T cells predicted better prognosis, implicating dysregulated differentiation and sustained activation in adverse outcomes. On-treatment, naïve CD21hi B cells, plasmablasts, activated CD8+ and central memory-like CD4+ T cells expanded, indicating de novo humoral responses and cytotoxic T cell invigoration. On-treatment, increased class-switched memory (IgG2+) B and activated T cells predicted improved survival, while persistent naïve and DN B cells were associated with poorer outcomes. Anti-PD-1 monotherapy expanded naïve (CD21hi) B cells and global T cells. Anti-PD-1/anti-LAG-3 (lymphocyte-activation gene 3) combination contracted memory B cells.

**Conclusions:**

Melanoma displays aberrant peripheral B and T cell activation, maturation and exhaustion, prominent in active disease. Treatment-induced class-switched B cells and T cell invigoration predict clinical benefit and naïve/DN B cells signify resistance. Coordinated B and T cell responses, especially recurrent extrafollicular B cell and exhausted/regulatory T cell states emerge as candidate indicators of outcome.

**WHAT IS ALREADY KNOWN ON THIS TOPIC**

- Despite the outstanding clinical success of checkpoint inhibitors (CPI) for the treatment of melanoma, there is a lack of predictive and dynamic biomarkers for response, toxicity and survival outcomes, representing an unmet clinical need. CPI efficacy likely depends on coordinated adaptive immune activation; however, integrated peripheral B and T cell signatures of clinical outcomes remain incompletely defined.

**WHAT THIS STUDY ADDS**

- Baseline dysregulation of circulating B cell and T cell populations consistent with extrafollicular maturation and exhaustion identifies patients at higher risk of toxicity and poor survival, revealing key immune vulnerabilities before treatment begins. During CPI treatment, the emergence of de novo B cell activation alongside activated cytotoxic T cells reveals effective adaptive immune response reinvigoration and indicates clinical outcomes. The coexistence of temporally-resolved adaptive immune correlates reveals dynamic biomarkers of treatment outcomes that have not previously been defined.

**HOW THIS STUDY MIGHT AFFECT RESEARCH, PRACTICE OR POLICY**

- Our findings reveal coexisting circulating adaptive immune signatures as candidates for prediction, risk stratification, and real-time monitoring of CPI benefit and toxicity, and support integrating standardized peripheral adaptive immune profiling to guide surveillance and early intervention.

## Introduction

 Checkpoint inhibitor (CPI) immunotherapy has revolutionized cancer treatment, causing a paradigm shift in treatment outcomes for melanoma, particularly for patients with advanced disease. Despite these successes, still only half of patients respond to treatment,^1^ and many patients develop immune-related adverse events (irAEs). These CPI-induced toxicities commonly target the skin, the gastrointestinal and endocrine systems,^2^ but vary significantly in the sites affected and their severity and frequently lead to long-term morbidity and can necessitate treatment interruption or discontinuation. For example, monotherapy of either nivolumab, ipilimumab and their combination can lead to high-grade irAEs in 23%, 28% and 59% of patients with advanced melanoma, respectively, with 38% of combination-treated patients forced to discontinue treatment.^3^ There are currently no predictive or dynamic biomarkers for both response and toxicity in standard clinical use, representing an unmet need to predict and monitor patient responses in melanoma.

The importance and prognostic significance of immune responses in cancer and strategies to advance clinical responses have largely focused on T cells.^4 5^ Tumor-infiltrating CD8+ cytotoxic T cells are considered the key prognostic immune population for stimulating antitumor immunity and for their roles in immunotherapy responses.^6–8^ Still, the circulating T cell reservoir has been increasingly studied to characterize expanded peripheral Tcell subsets and to identify exhausted T cell phenotypes in the peripheral blood. These circulating immune signatures often precede or mirror intratumoral dynamics and may associate with CPI response.^9^

In recent years, there has been a growing interest in the impact of the humoral compartment on antitumor immunity and their contribution to effective CPI responses.^10–15^ Particularly, B cells in organized lymphoid aggregates akin to tertiary lymphoid structures within tumors may undergo maturation and synergize with follicular T helper cells (Tfh) and dendritic cells to promote antitumor responses.^10 12 16 17^ However, previous studies have indicated Janus-faced roles of B cells in melanoma.^10 11 18 19^ Unlike their T cell counterparts, regulatory B cells (Bregs) do not express a specific transcription factor, are not associated with a specific lineage and span the B cell differentiation spectrum,^20^ suggesting functional and phenotypic plasticity in the context of cancer. Bregs are typically characterized by their expression of transforming growth factor (TGF)-ß, interleukin (IL)-10, programmed cell death protein 1 (PD-1) and programmed death ligand 1 (PD-L1).^21–27^ Recent evidence has suggested prominent roles of Bregs in patients with melanoma: circulating and tumor-resident TGF-ß+ B cells promote Forkhead box protein P3 (FoxP3)+ regulatory T cell (Treg) induction in a TGF-ß-dependent manner^20^; IL-10+plasmablasts and alternatively-activated double negative (DN, IgD−CD27−) B cells are enriched in melanoma compared with healthy circulation^18^; and a Breg subset (IgG4+CD49b+CD73+) has been reported to promote angiogenesis and inflammation via production of vascular endothelial growth factor (VEGF).^28^

Tumor-infiltrating B and T cells have been well characterized in melanoma.^10 29–33^ However, circulating humoral/adaptive immune signatures may also be valuable for predicting treatment responses and are accessible for dynamic monitoring of patients.^29 34–38^ Baseline^18^ and on-treatment changes in B cell phenotypes were found to correlate with irAE induction,^34^ and the ratio of exhausted CD8 T cell reinvigoration to tumor burden is reported as a predictor of clinical response to anti-PD-1 therapy.^37^ Baseline B cell phenotypes and autoantibody levels may also predict lack of toxicity to anti-PD-1,^18^ on-treatment changes in B cell frequencies predict irAE onset^34^ and naïve CD4 T cell abundance at baseline have been associated with severe irAE development following combination CPI.^39^

Here, we hypothesize that the coexistence of naïve, regulatory and immunosuppressive B and T cell phenotypes reflects a state of adaptive immune dysregulation, not previously interrogated simultaneously across both humoral and cellular compartments in the context of CPI. Such signatures may be identifiable both a priori and longitudinally during therapy and may inform prediction and real-time monitoring of immunotherapy response. To test this, we employ high-dimensional profiling by cytometry time-of-flight (CyTOF) to comprehensively characterize circulating B and T cell subsets from patients with melanoma at baseline and during treatment. By integrating immune profiling data with clinical parameters and treatment outcomes, we aim to define parallel humoral and cellular immune features of therapeutic response. Specifically, we delineate baseline immune landscapes in patients relative to healthy volunteers, identify predictive immune signatures of subsequent treatment response, and leverage longitudinal sampling to characterize dynamic changes in B and T cell phenotypes during CPI and their relationship to patient outcomes.

## Materials and methods

### Human sample collection

Whole blood samples were collected in 10 mL EDTA blood collection tubes. Peripheral blood mononuclear cell isolation and cryopreservation were performed as previously described^18 29 40 41^ (online supplemental materials).

### Patient characteristics

Blood samples were collected from 24 patients with stage II-IV cutaneous melanoma who were CPI immunotherapy-naive and 25 healthy volunteers (online supplemental tables S1-2). Pre-treatment (baseline) peripheral blood samples were collected prior to the start of CPI immunotherapy (n=24), and matched patient on-treatment samples were collected at two timepoints during immunotherapy; Timepoint A (after 6-8 weeks, n=18) and Timepoint B (after 12 weeks, n=12) (online supplemental figure S1). Patients received either anti-PD1 monotherapy (n=14), anti-PD1/LAG3 (lymphocyte-activation gene 3) combination (n=6) or anti-PD1/CTLA4 combination (n=4) (online supplemental table S1).

IrAE grading was reported according to European Society for Medical Oncology (ESMO) clinical guidelines applied by clinical oncologists involved in patient care. To define toxicity in this study, patients were assigned as “no toxicity”, “high-grade toxicity” or “all toxicity”. “High-grade” toxicity was defined as grade 3 or higher according to ESMO and “all toxicity” encompassed all patients with toxicity (grade 1–4 irAE). We used the Strengthening the Reporting of Observational Studies in Epidemiology (STROBE) case–control checklist.^42^

### Suspension mass cytometry and cluster analysis using the FlowSOM clustering algorithm

CyTOF of B and T cells was conducted to facilitate high-dimensional immunophenotyping. The gating strategy to isolate pure CD19+ and CD3+ populations is shown in online supplemental figure S2. A previously described marker panel was adapted to include T cell markers and immunoglobulin isotypes (online supplemental table S3).^18 41^ Cell staining, data acquisition and clustering analysis are detailed in online supplemental materials.

### Statistical analysis

#### CyTOF analyses of B and T cell subsets

Statistical analyses of cluster expression between populations were performed using two models. We conducted differential abundance (DA) testing using the diffcyt and edgeR packages, a computational generalized linear mixed model that compensates for sample size variability, accounts for patient–patient variability and has integrated false discovery rate adjustment using the Benjamini-Hochberg method, as previously described.^43 44^ In addition, paired longitudinal samples between two time points were assessed using the Wilcoxon rank-sum test or paired t-test depending on normality distribution (using the Shapiro-Wilk normality test), and one-way analysis of variance (ANOVA) was used for paired analysis between three time points.

#### Survival analysis

Survival analyses were conducted using the number of days between the first dose of checkpoint inhibitor therapy as day 0, and the endpoint of “overall survival” (OS) and “event-free survival” (EFS). The endpoint for OS was determined as death due to melanoma. The endpoint for EFS was determined by progression on treatment (as determined by surveillance scans detailed in clinical notes) for active metastatic patients with measurable disease at baseline, or death by melanoma for adjuvant/resected disease patients. The median follow-up was 643 days for OS (death) and 605 days for EFS (event; progression or death); the majority of patients had a follow-up time of <1,000 days, and just four patients had an extended follow-up of >1,000 days due to time of sample collection. Patient survival and progression information is detailed in online supplemental table S4. B and T cell cluster frequencies were treated as continuous variables and dichotomized for Kaplan-Meier survival analysis. Optimal cut-off points of “high” versus “low” abundance of each cluster were determined by using receiver operating characteristic (ROC) analysis and the Youden Index to maximize sensitivity and specificity, using the cutpointR package. ROC analysis used event status as the binary outcomes (death for OS and progression for EFS) and patients were stratified into high and low groups based on the optimal cut-off. Plots with fewer than six events per group (quartile) were excluded from the analysis. Kaplan-Meier plots were generated using GraphPad Prism and statistical significance was determined by using the log-rank test. Multivariate Cox proportional hazard models were used to supplement the survival analysis.

## Results

### Enrichment of naïve/DN2 B cells and plasmablasts and activated cytotoxic and DN T cells reflect adaptive immune remodeling in patients with melanoma

To delineate systemic lymphocyte profiles in patients with melanoma, we conducted high-dimensional profiling of circulating B (CD19+) and T (CD3+) cells from treatment-naïve patients with melanoma (n=24, stage II/III/IV) and healthy volunteers (n=25). We used a 45-marker CyTOF panel, applying FlowSOM-based clustering, high-dimensional embeddings (t-distributed Stochastic Neighbor Embedding, t-SNE/Uniform Manifold Approximation and Projection**,** UMAP), and generalized linear mixed-model DA analyses.

**Figure 1:**
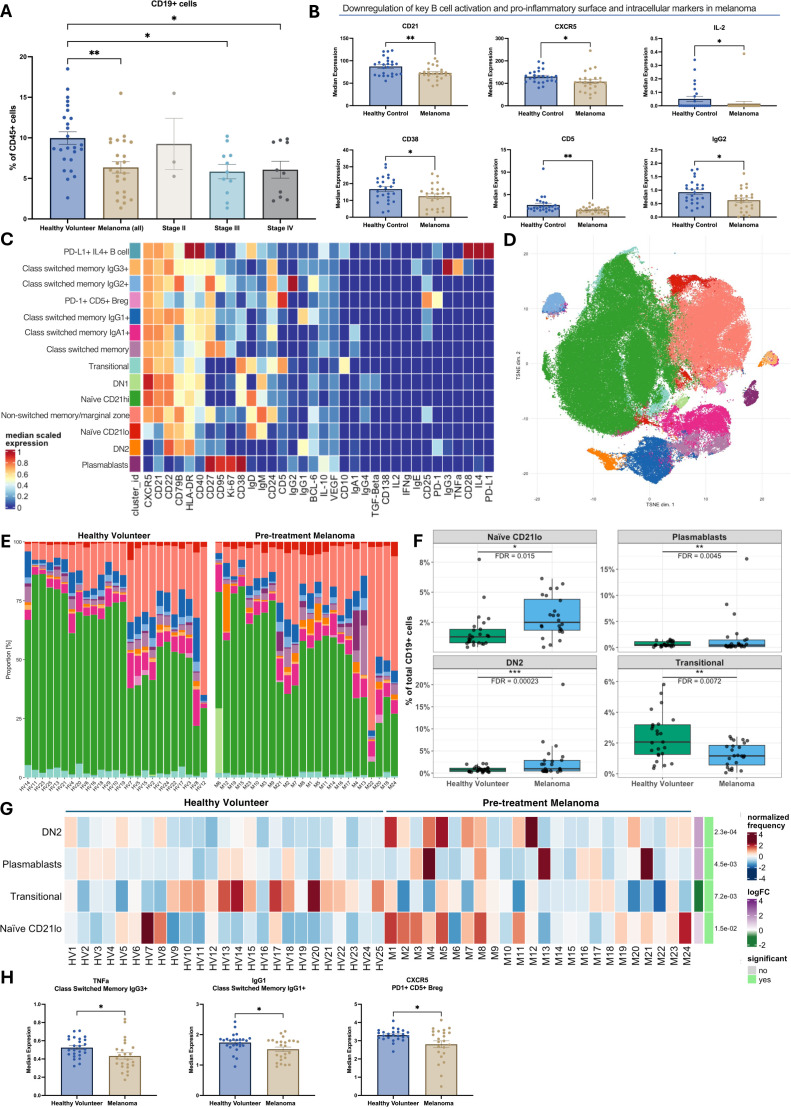
Deep phenotypic characterization of B cells by CyTOF analyses reveals naïve, immunosuppressive and alternatively-activated circulating humoral immunity enriched in patients with melanoma (n=24) compared with healthy volunteers (n=25). (**A**) Relative abundance of total B cells (CD19+) as a proportion of CD45+ cells in healthy volunteers and patients with melanoma (divided by stage; melanoma (all), n=24; stage 2, n=3; stage 3, n=11; stage 4, n=10) (one-way ANOVA with Tukey’s multiple comparisons test). (**B**) Differential expression of surface and intracellular markers between melanoma and healthy volunteer B cells (unpaired t-test or Mann-Whitney depending on normality distribution), based on scaled median marker expression of each marker. (**C**) Cluster heatmap for the B cell dataset of the 14 merged clusters, showing the median scaled expression (scaling at 0.01% quartile) of the 34 markers used for B cell clustering (FlowSOM algorithm). (**D**) t-SNE of the B cell dataset showing the 14 merged B cell clusters. (**E**) Abundance bar chart representing proportions of merged clusters per donor between healthy volunteers and patients with melanoma. (**F**) Comparisons of relative cluster proportions between HV and patients with melanoma B cell clusters (diffcyt DA test, FDR corrected with Benjamini-Hochberg method, significant adjusted p value<0.05). (**G**) DA test showing significantly differentially expressed B cell clusters between HVs and patients with melanoma. Normalized frequency indicates the relative abundance of each cluster per sample, and LogFC indicates the change in frequency of each cluster between the two cohorts. Green bars indicate statistically significant differential expression after multiple test correction. (**H**) Differential expression of surface and intracellular markers on specific B cell subsets in patients with melanoma and HVs (Welch’s t-test for normally distributed data). Data shown as mean±SEM. *p<0.05; **p<0.01, **p<0.001, ***p<0.0001. ANOVA, analysis of variance; Breg, regulatory B cell; CyTOF, cytometry time-of-flight; DA, differential abundance; FDR, false discovery rate; LogFC, log fold change; PD-1, programmed cell death protein 1; PD-L1, programmed death ligand 1; TNF, tumor necrosis factor; t-SNE, t-distributed Stochastic Neighbor Embedding; UMAP, Uniform Manifold Approximation and Projection**.**

Lower overall CD19+ B cell frequency (figure 1A) and broad downregulation of activation/trafficking (CD38, CD21, CD5, CXCR5) molecules, pro-inflammatory cytokines (IL-2, although at low levels in freshly isolated B cells), and immunoglobulins (IgG2) were observed on patient B cells (scaled median marker expression) (figure 1B). Reduced CD19+ B cell frequency is exaggerated in advanced (stage III/IV) disease (figure 1A). An unsupervised FlowSOM algorithm using 34 markers was used to generate 20 B cell metaclusters (online supplemental figure S3A), followed by manually merging into 14 canonical B cell subsets as conducted previously (details of marker expression and cluster merging in online supplemental figure S3A,B).^18 29 44^ Merged clusters were visualized by heatmap, t-SNE and proportional abundance (figure 1C–E). Relative abundance and a generalized linear mixed model DA analysis showed an enrichment of circulating naïve CD21lo and DN2 (IgD−CD27−CD21−CXCR5−) B cell subsets and plasmablasts (expressing IL-10/CD95), with reduced transitional B cells in melanoma (figure 1F–G; DA at unsupervised metacluster level in online supplemental figure S3C); enrichment in naïve CD21lo and reduction in transitional B cells is seen between healthy volunteers (HV) and stage III disease (online supplemental figure S3D). Furthermore, class switched memory B cells and PD-1+ B cells feature lower levels of mature/proinflammatory (tumor necrosis factor-α and IgG1) and cell trafficking (CXCR5) molecules (figure 1H).

**Figure 2:**
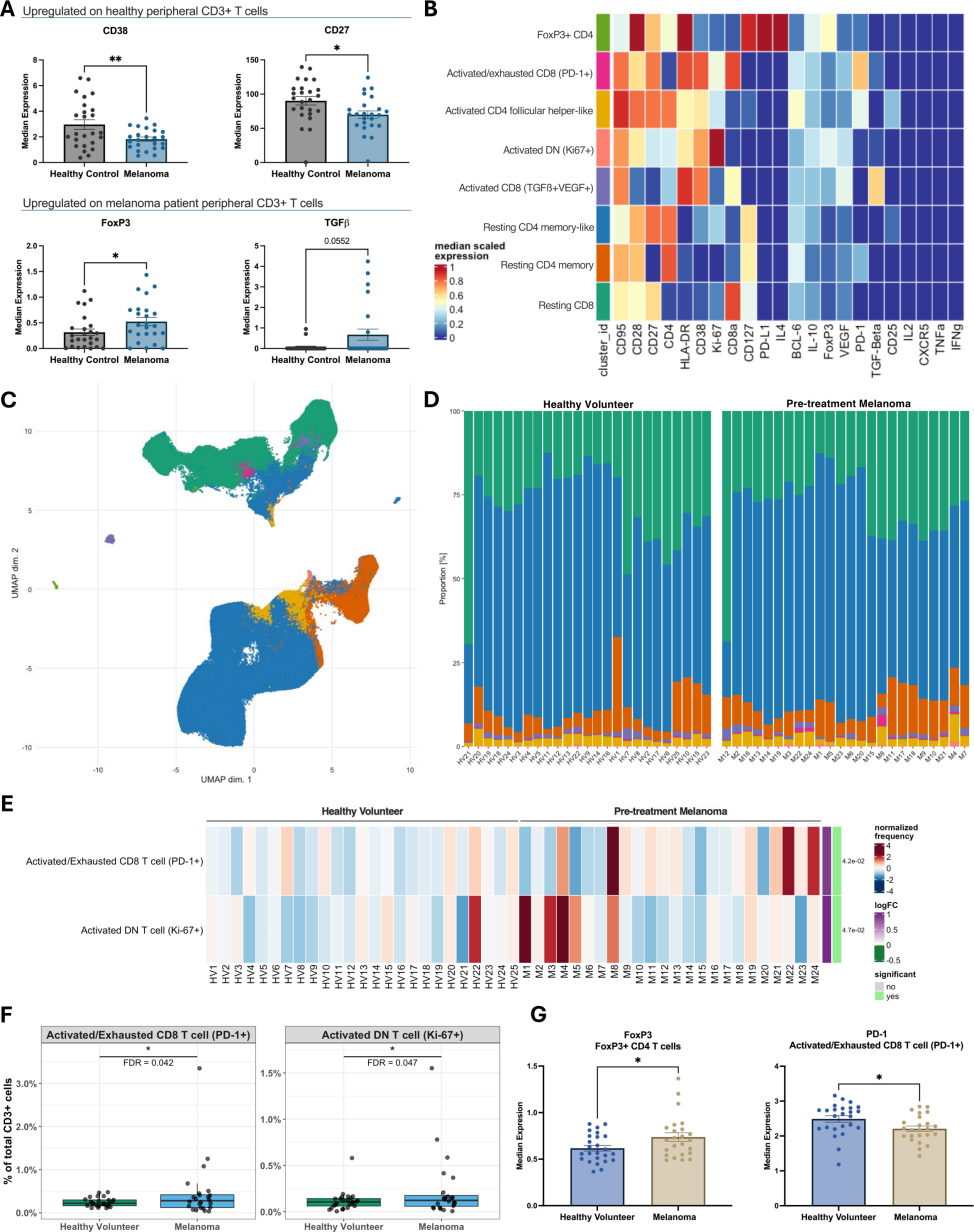
Deep phenotypic characterization of T cells using CyTOF reveals enrichment of proliferating and exhausted T cells and regulatory features in melanoma (n=24) circulation compared with healthy volunteers (n=25). (**A**) Differential expression of surface and intracellular markers between melanoma and HV T cells (unpaired t-test or Mann-Whitney depending on normality distribution), based on scaled median marker expression. (**B**) Cluster heatmap for the T cell dataset, showing the median scaled expression of the 34 markers (scaling at 0.01% quartile) used for T cell clustering (FlowSOM algorithm) into eight distinct clusters. (**C**) UMAP of the T cell dataset showing the eight merged T cell clusters. (**D**) Abundance bar chart showing proportions of merged T cell clusters per donor between healthy volunteers and patients with melanoma. (**E**) DA test showing significantly differentially expressed T cell clusters between HVs and patients with melanoma. Normalized frequency indicates the relative abundance of each cluster per sample, and LogFC indicates the change in frequency of each cluster between the two cohorts. Green bars indicate statistically significant differential expression after multiple test correction. (**F**) Comparisons of relative cluster proportions between HV and patients with melanoma T cell clusters (diffcyt DA test, FDR corrected with Benjamini-Hochberg method, significant adjusted p value<0.05). (**G**) Differential expression of surface and intracellular markers on specific T cell subsets in patients with melanoma and HVs (Mann-Whitney test for non-normally distributed data). Data shown as mean±SEM. *p<0.05; **p<0.01, **p<0.001, ***p<0.0001. CyTOF, cytometry time-of-flight; DA, differential abundance; DN, double negative; FDR, false discovery rate; LogFC, log fold change; PD-1, programmed cell death protein 1; TGF, transforming growth factor; VEGF, vascular endothelial growth factor; Ki-67, proliferation marker protein-67; UMAP, Uniform Manifold Approximation and Projection map; HV, healthy volunteer; FoxP3, Forkhead box protein P3.

In the T cell compartment, overall CD3+ frequency is unchanged in healthy versus melanoma peripheral blood (online supplemental figure S4A); however, decreased activation/memory (CD38, CD27) and increased regulatory (FoxP3, TGF-β) markers are expressed on total T cells from patients (figure 2A). Unsupervised clustering using 22 markers generated 8 T cell subsets (clustering/visualization: figure 2B–D), with activated T cell subsets defined as HLA-DR+CD38+, as previously described^45^ within the confinements of our panel. DA analyses identified enrichment of activated/exhausted cytotoxic (CD8, PD-1+) and activated DN (CD4−CD8−Ki67+(proliferation marker protein-67)) T cell clusters in melanoma (figure 2E,F), with higher FoxP3 in FoxP3+CD4 T cells and lower PD-1 on activated CD8 PD-1+ T cells (figure 2G). Spearman rank correlations showed no significant impact of age on the frequency of significantly enriched cell types in patients with melanoma (online supplemental figure S4B).

Together, these data indicate that treatment-naïve melanoma is characterized by systemic remodeling of adaptive immunity, including increased naïve/DN2 and IL-10+ plasmablast-like B cell states and expanded activated cytotoxic, DN T cell populations and regulatory T cell features, reflecting a broadly dysregulated circulating adaptive immune environment that becomes more pronounced with advancing disease.

### Enrichment of naïve/transitional B cells and exhausted/immunosuppressive T cells predicts disease activity, toxicity susceptibility to CPI and survival outcomes

To define how baseline adaptive B and T cell immunity relates to active disease status, to subsequent toxicity on CPI therapy and to clinical outcomes following CPI, we analyzed circulating B and T cells from patients (n=24) who subsequently received CPI (clustering/visualization: online supplemental figure S5A-D). We compared immune features between patients with active, unresected disease (n=15) versus resected disease (n=9) at baseline. Furthermore, we assessed immune signatures in patients stratified by subsequent treatment-related toxicity status and survival outcomes.

**Figure 3:**
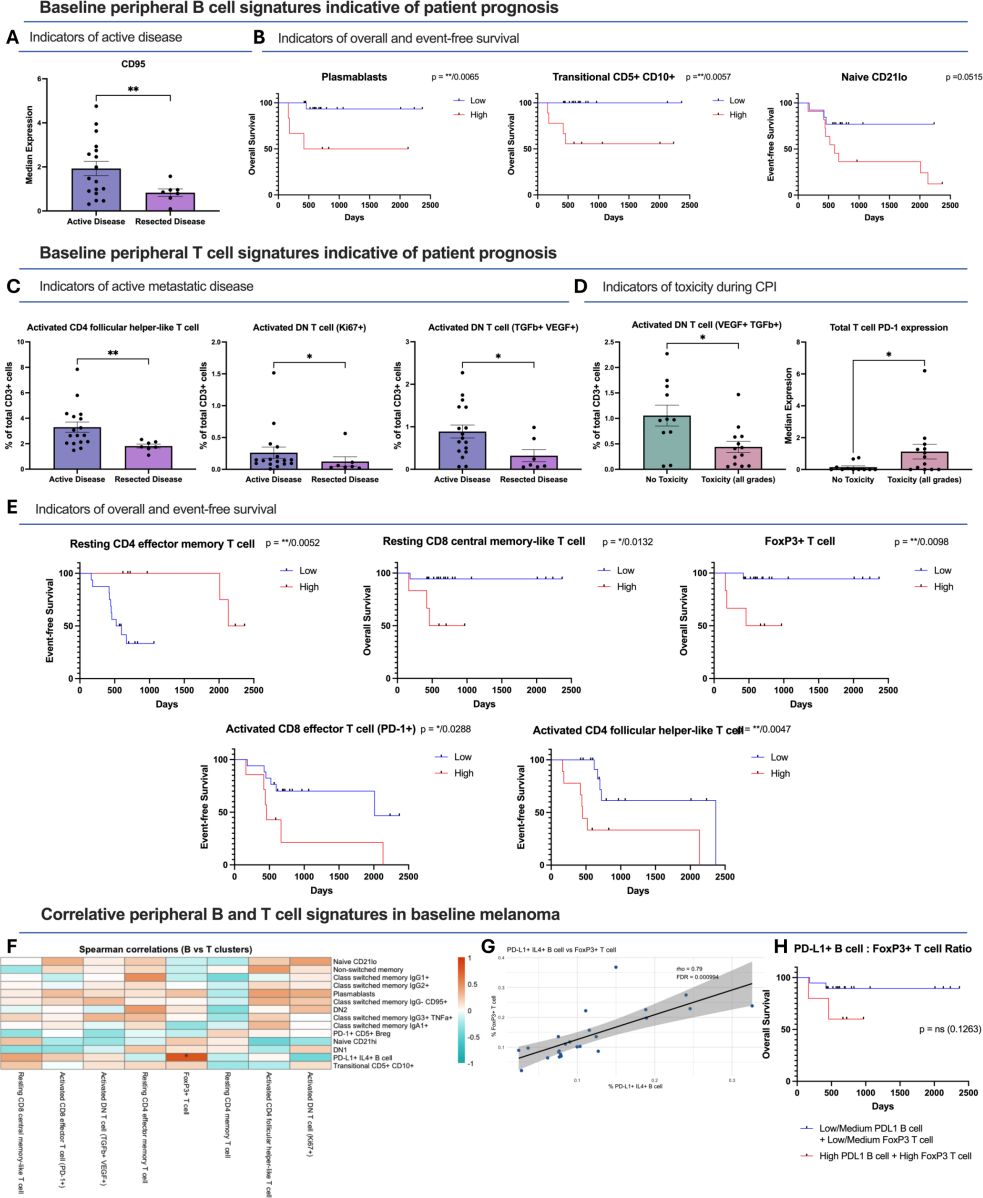
B and T cell signatures predict and correlate with disease state, toxicity and survival outcomes in patients with melanoma at baseline before starting immunotherapy (n=24). (**A**) Comparison of scaled median expression of markers on B cells between patients with active, unresected disease (n=17) versus resected disease (n=7) at baseline (Welch’s t-test for normally distributed data). (**B**) Kaplan-Meier curves for baseline B cell phenotypes that significantly predict overall and event-free survival (n=24) (log-rank Mantel-Cox test). Overall survival: Plasmablasts “low” n=18 and “high” n=6, transitional B cells “low” n=15 and “high” n=9. Event-free survival: naïve CD21lo B cells “low” n=13 and “high” n=11. (**C**) Comparisons of relative cluster abundance of T cell subsets that are differentially expressed between “active disease” (n=17) and “resected disease” (n=7) groups at baseline (Mann-Whitney). (**D**) Comparisons of relative cluster abundance of T cell subsets that are differentially expressed between “no toxicity” (n=11) and “toxicity” (all grades, n=13) groups at baseline, and comparison of median expression of markers on T cells between patients that subsequently developed toxicity (Mann-Whitney). (**E**) Kaplan-Meier curves for baseline T cell phenotypes that significantly predict overall and event-free survival (log-rank Mantel-Cox test). Overall survival: resting CD8 central memory-like T cell “low” n=18 and “high” n=6, FoxP3+ T cell “low” n=18 and “high” n=6. Event-free survival: resting CD4 effector memory T cell “low” n=16 and “high” n=8, activated CD8 effector T cell (PD-1+) “low” n=17 and “high” n=7 and activated CD4 follicular helper-like T cell “low” n=15 and “high” n=9. (**F**) Spearman correlation coefficient heatmap for paired analysis of B and T cell clusters between patients (n=24) at baseline. (**G**) Spearman correlation showing the significant positive correlation between PD-L1+ B cells and FoxP3+ T cells in patients at baseline (n=24). (**H**) Kaplan-Meier curve showing the association between high PD-L1+ B cell: FoxP3+ T cell ratio and overall survival (log-rank Mantel-Cox test). Data shown as mean±SEM. *p<0.05; **p<0.01, **p<0.001, ***p<0.0001. CPI, checkpoint inhibitor; FoxP3, Forkhead box protein P3; Ki-67, proliferation marker protein-67; PD-1, programmed cell death protein 1; PD-L1, programmed death ligand 1; TGF, transforming growth factor; VEGF, vascular endothelial growth factor.

B cells in treatment-naïve patients with active metastatic disease showed significantly higher expression of the death receptor and regulatory marker CD95 compared with B cells in resected disease, consistent with chronic antigen exposure and immune stimulation (figure 3A). Prior to treatment, enrichment of B cell immune features known to be associated with extrafollicular pathways predicted survival outcomes: plasmablasts and transitional (CD5+CD10+) B cells correlated with reduced OS, and elevated naïve CD21lo B cells were associated with reduced EFS (figure 3B). Multivariate Cox proportional hazard analysis indicated enrichment of naïve CD21lo B cells at baseline predicts reduced EFS (S5E). These findings reflect a skew in favor of immature subsets with Breg potential.

Three activated circulating T cell subsets, Tfh-like cells (CD4+BCL6+PD-1^med^), proliferating DN (CD4−CD8−Ki67+) T cells and immunosuppressive DN (CD4−CD8−TGFβ+VEGF+) T cells were significantly enriched in patients with active versus resected disease (figure 3C), consistent with T cell responses to chronic antigenic stimulation. Stratification by toxicity status to CPI revealed higher baseline frequencies of activated/immunosuppressive DN T cells (VEGF+TGF-β+) in patients who did not develop irAEs during CPI treatment (n=11), while PD-1 expression was significantly elevated on T cells from patients who subsequently experienced grade 1–4 irAEs (n=13) (figure 3D). Baseline enrichment of resting central memory-like CD8+and FoxP3+ T cells was associated with reduced OS and activated/exhausted CD8+ (PD-1+) T cells and CD4 follicular helper-like T cells correlated with reduced EFS (figure 3E). While high frequencies of resting CD4 effector memory T cells were associated with improved EFS (figure 3E). Multivariate Cox analysis complemented these findings; baseline enrichment of resting CD8 central memory-like and activated CD8 (PD-1) T cells correlated with reduced OS and activated DN (TGF-β+VEGF+) T cells associated with reduced EFS (online supplemental figure S5E). These data indicate that insufficiently activated, exhausted or immunoregulatory T cell states prior to treatment may limit effective cytotoxic antitumor functions triggered by immunotherapy.

Paired analyses of baseline circulating B and T cell profiles revealed a significant correlation between patients with high frequencies of PD-L1+ B cells and FoxP3+ T cells (Spearman rank correlation, figure 3F–G), but this was not associated with prognosis (figure 3H).

In summary, prior to treatment, adaptive immune signatures across both B cell and T cell compartments reflect underlying disease activity and carry predictive relevance for toxicity risk and clinical outcomes in CPI-treated patients. Patients with higher proportions of naïve, transitional, regulatory, or exhausted B and T lymphocyte states consistently associate with poorer outcomes on CPI, whereas CD4 effector memory T cell readiness reflects a more effective antitumor immune baseline. These highlight that reduced humoral maturation and an immunoregulatory T cell bias at treatment initiation may limit effective antitumor responses to checkpoint blockade.

### Checkpoint inhibitors drive de novo circulating naïve B cell responses and activated cytotoxic T cells on treatment correlating with patient outcomes

We performed longitudinal CyTOF profiling of circulating lymphocytes from patients with melanoma receiving CPI therapy at baseline (pretreatment, n=24), Time point A (6–8 weeks, n=18) and Time point B (12 weeks, n=12). Paired samples enabled intrapatient comparisons across treatment.

**Figure 4:**
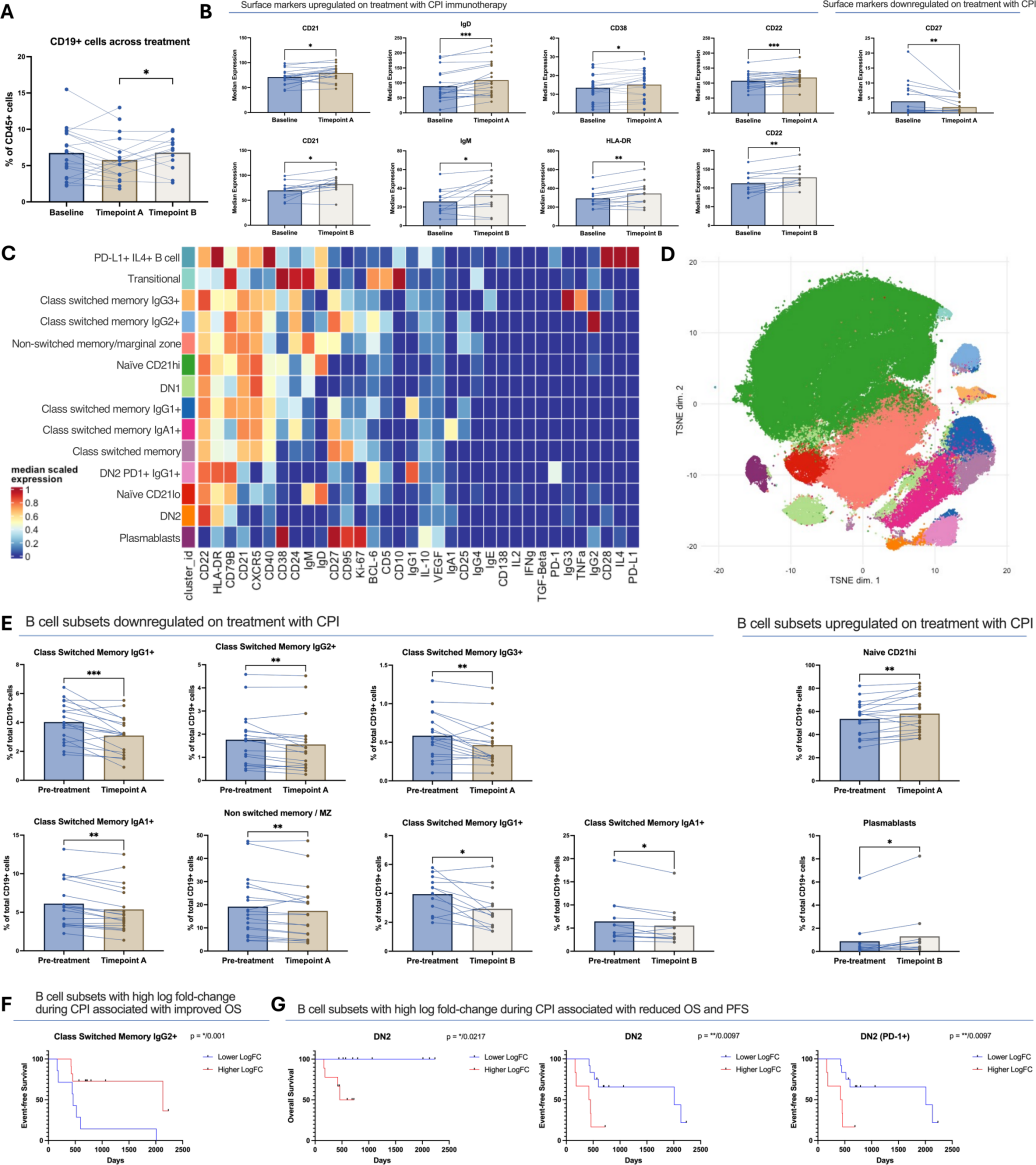
Checkpoint inhibitor immunotherapy stimulates humoral immune responses via a de novo activation of naïve B cells and plasmablasts. (**A**) Relative abundance of total B cells (CD19+) as a proportion of CD45+cells in paired patients with melanoma samples across three time points on treatment (baseline—Time point A n=18, Time point A—Time point B n=12) (mixed effects analysis with Holm-Sidak’s multiple correction test). (**B**) Differential expression of surface markers (scaled median expression) on patients with melanoma B cells between baseline and Time point A (n=18) and Time point B (n=12) (paired t-test or Wilcoxon test depending on normality distribution (Shapiro-Wilk)). (**C**) Cluster heatmap for the B cell dataset showing the 14 merged clusters, showing the median scaled expression (scaling at 0.01% quartile) of the 34 markers used for B cell clustering (FlowSOM algorithm). (**D**) t-SNE of the patients with melanoma B cell dataset showing the 14 merged B cell clusters. (**E**) Comparisons of relative cluster proportions between paired patients with melanoma samples of B cell clusters at baseline and Time point A (n=18) and Time point B (n=12) (paired t-test or Wilcoxon depending on normality distribution). (**F**) Kaplan-Meier analyses of log fold change of B cell subsets between baseline and Time point A of CPI that predict favorable overall and event-free survival (n=18). Class switched memory IgG2+ “low” n=7 and “high” n=11. (**G**) Kaplan-Meier analyses of log fold change of B cell subsets between baseline and Time point A of CPI that predict reduced overall and event-free survival (n=18) (log-rank Mantel-Cox test). Overall survival: DN2 “low” n=9 and “high” n=9. Event-free survival: DN2 “low” n=12 and “high” n=6, DN2 (PD-1+) “low” n=12 and “high” n=6. Data shown as mean±SEM. *p<0.05; **p<0.01, **p<0.001, ***p<0.0001. CPI, checkpoint inhibitor; DN, double negative; IL, interleukin; LogFC, log fold change; OS, overall survival; PD-1, programmed cell death protein 1; PD-L1, programmed death ligand 1; PFS, progression-free survival; t-SNE, t-distributed Stochastic Neighbor Embedding.

CD19+ B cell abundance within the circulating CD45+ compartment increased significantly from Time point A to Time point B in paired samples (figure 4A). Compared with baseline, CD21, CD22, CD38 and IgD were increased at Time points A (n=18, paired); CD21, IgM, HLA-DR and CD22 were elevated by Time point B (n=12, paired); while the memory marker CD27 was reduced at Time point A (figure 4B).

FlowSOM identified 20 metaclusters, merged into 14 B cell subsets (online supplemental figure S6A,B), with per-donor distributions (online supplemental figure S6C) and visualizations by cluster heatmap and t-SNE (figure 4C,D). Relative frequency of paired samples showed early reduction (6–8 weeks) of class-switched memory IgG1+, IgG2+, IgG3+, IgA1+, and non-switched memory B cell clusters, with persistent decreases in IgG1+ and IgA1+ memory B cells at 12 weeks (figure 4E). Conversely, naïve CD21hi B cells were increased at Time point A and plasmablasts (IL-10+CD95+) were increased at Time point B (figure 4E), indicating CPI-induced de novo naïve B cell compartment induction.

For clinical correlates, high positive log fold change (LFC) from baseline to Time point A (n=18) of IgG2+ class switched memory B cells associated with improved EFS (figure 4F), whereas expansion of DN2 B cell subsets predicted reduced OS and EFS (figure 4G). Multivariate Cox analysis supported these associations; high LFC of class switched memory IgG2+ B cells indicated improved OS and EFS, and DN2 subsets correlated with reduced prognosis (online supplemental figure S6D). Enrichment of Th2-biased IL-4+PD-L1+ B cells at Time point A during treatment correlated with improved OS, while high frequencies of naïve CD21lo and DN subsets on treatment predicted reduced OS and EFS (online supplemental figure S6E).

**Figure 5:**
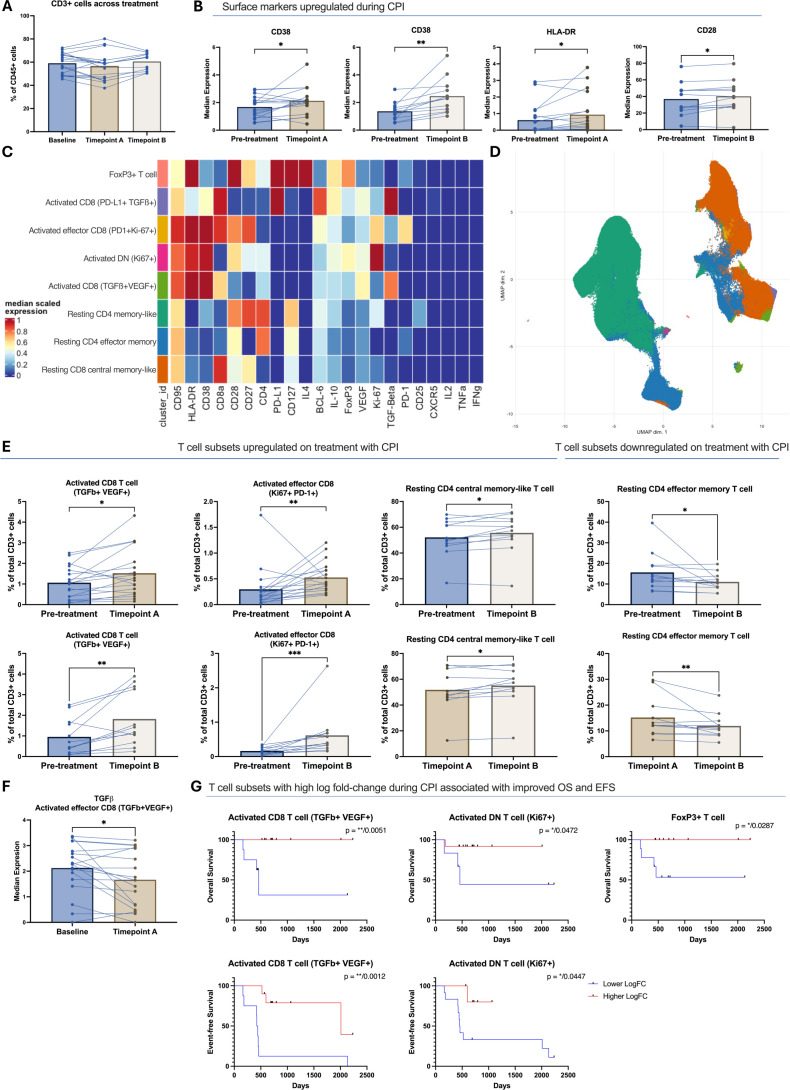
Checkpoint inhibitor immunotherapy stimulates T cell immunity to promote activated cytotoxic and resting memory CD4 T cells that are associated with improved prognosis. (**A**) Relative abundance of total T cells (CD3+) as a proportion of CD45+ cells in paired patients with melanoma samples across three time points on treatment (baseline—Time point A n=18, Time point A—Time point B n=12) (mixed effects analysis with Holm-Sidak’s multiple correction test). (**B**) Differential expression of surface markers (scaled median expression) on patient with melanoma T cells between baseline and time point A (n=18) and time point B (n=12) (paired t-test or Wilcoxon test depending on normality distribution (Shapiro-Wilk)). (**C**) Cluster heatmap for the T cell dataset showing the eight clusters, showing the median scaled expression (scaling at 0.05% quartile) of the 22 markers used for T cell clustering (FlowSOM algorithm). (**D**) UMAP of the patients with melanoma T cell dataset showing the eight T cell clusters. (**E**) Comparisons of relative cluster proportions between paired patients with melanoma samples of T cell clusters at baseline and time point A (n=18) and time point B (n=12) (paired t-test or Wilcoxon depending on normality distribution). (**F**) Differential expression of markers on specific T cell subsets in patients with melanoma at baseline and on treatment at time point A (n=18) (paired t-test or Wilcoxon depending on normality distribution). (**G**) Kaplan-Meier analyses of log fold change of T cell subsets, between baseline and time point A (n=18) of CPI, that predict overall and event-free survival (log-rank Mantel-Cox test). Overall survival: activated CD8 T cell (TGF-β+VEGF+) “low” n=8 and “high” n=10, activated DN T cell (Ki67+) “low” n=6 and “high” n=12 and FoxP3+ T cell “low” n=9 and “high” n=9. Event-free survival: activated CD8 T cell (TGF-β+VEGF+) “low” n=8 and “high” n=10 and activated DN T cell (Ki67+) “low” n=12 and “high” n=6. Data shown as mean±SEM. *p<0.05, **p<0.01, **p<0.001, ***p<0.0001. CPI, checkpoint inhibitor; DN, double negative; EVS, event-free survival; FoPX3, Forkhead box protein P3; Ki-67, proliferation marker-67; LogFC, log fold change; OS, overall survival; PD-1, programmed cell death protein 1; PD-L1, programmed death ligand 1; TGF, transforming growth factor; UMAP, Uniform Manifold Approximation and Projection; VEGF, vascular endothelial growth factor.

The relative abundance of CD3+ T cells within the circulating CD45+ compartment did not change significantly across time points in paired samples (figure 5A). Nevertheless, total T cell activation increased: CD38 and HLA-DR were upregulated on patient T cells between baseline and Time point A (n=18, paired); CD38 and CD28 were upregulated between baseline and Time point B (n=12, paired) (figure 5B).

FlowSOM clustering defined eight T cell clusters (proportions, online supplemental figure S7A; heatmap, figure 5C; UMAP, figure 5D). Paired analyses showed proportional increases in activated CD8+ (TGF-β+VEGF+) and activated effector CD8+ (PD-1+Ki67+) T cells from baseline to Time point A (n=18) and Time point B (n=12) (paired figure 5E, unpaired online supplemental figure S7B,C), and expansion of resting central memory-like CD4+ T cells from baseline to Time point B and from Time point A to Time point B (n=11) (figure 5E). Resting effector memory CD4+ T cells reduced from baseline to Time point B and Time point A to Time point B (figure 5E). Reduced expression of the immunosuppressive cytokine TGF-β on activated CD8 (TGF-β+VEGF+) T cells was observed during treatment (figure 5F).

Survival analyses using LFC (baseline to Time point A, n=18) showed associations between higher on-treatment expansion of activated CD8+ (TGF-β+VEGF+), activated DN (Ki67+) and FoxP3+ T cells with improved OS (figure 5G). Increased activated CD8+ (TGF-β+VEGF+) and activated DN (Ki67+) T cells also associated with improved EFS (figure 5G). Multivariate Cox analyses complemented these findings; high LFC during treatment of activated DN (Ki67+) correlated with improved OS/EFS and activated CD8+ (TGF-β+VEGF+) predicted better OS (online supplemental figure S7D). On-treatment enrichment of activated DN (Ki67+) T cells predicted improved OS and EFS (online supplemental figure S7E).

These data demonstrate that CPI treatment orchestrates coordinated remodeling of the peripheral adaptive immune compartment: contraction of class-switched memory B cells with de novo activation of naïve B cells and emergence of plasmablasts, alongside heightened T cell activation with expansion of effector CD8+, central memory-like CD4+, and proliferative/immunosuppressive T cell subsets. The magnitude and direction of these on-treatment shifts stratify OS and EFS, nominating early B cell and T cell dynamics as putative biomarkers of CPI efficacy in melanoma.

### Expansion of circulating naïve (CD21hi) B cell and effector CD8+ T cells during anti-PD-1 monotherapy while anti-PD-1 combination with anti-LAG-3 reduces circulating memory B cells

To compare how distinct CPI regimens shape circulating adaptive immunity, we analyzed the same matched longitudinal samples at baseline and Time point A (6–8 weeks; n=18) and Time point B (12 weeks; n=12), stratifying patients receiving anti-PD-1 monotherapy (n=11) and anti-PD-1/LAG-3 (n=6) combination.

**Figure 6:**
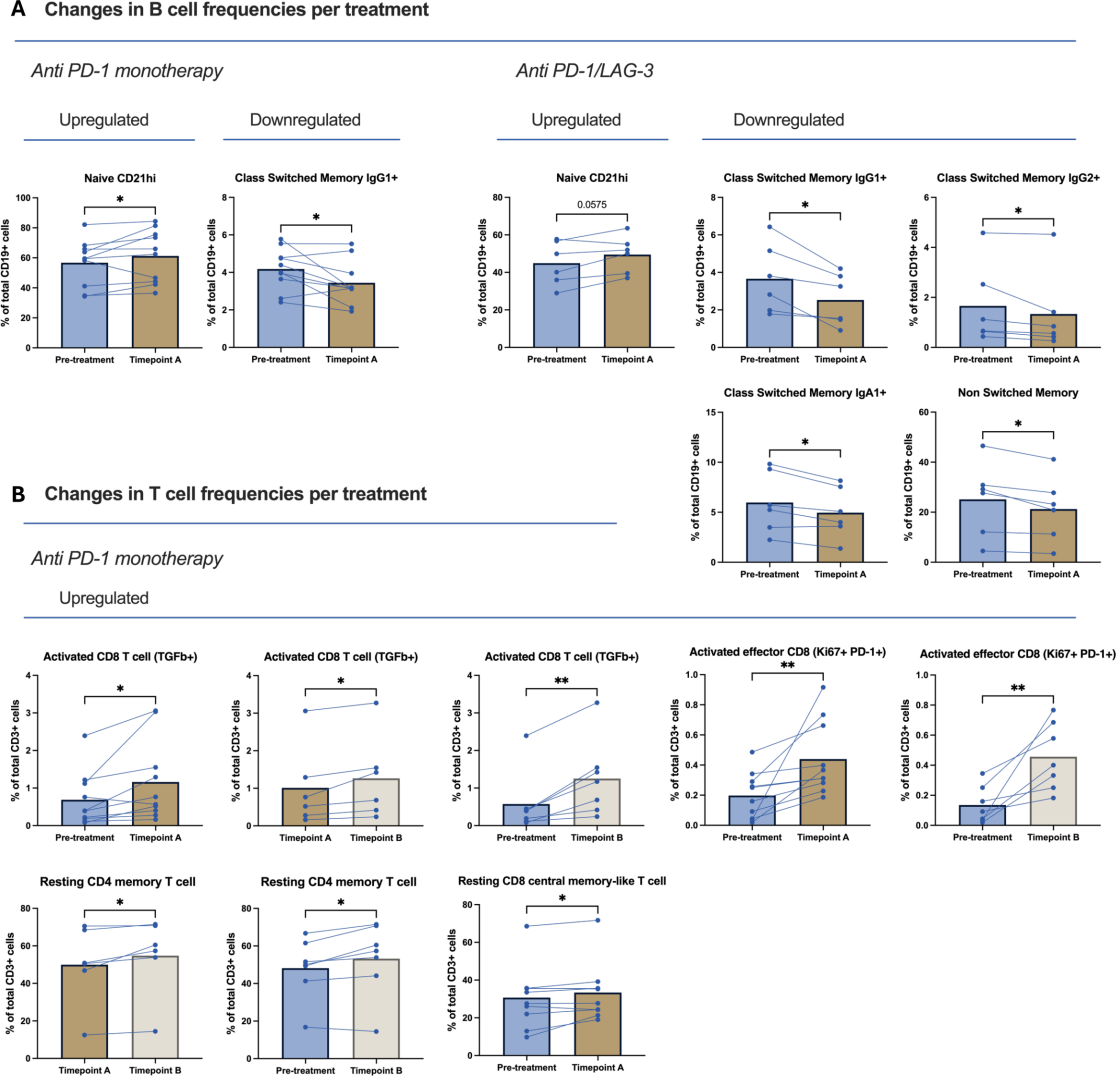
Anti-PD-1 monotherapy stimulates the proportional expansion of activated naïve B cells and global T cell populations while anti-PD-1/LAG-3 combination retracts circulating memory B cells. (**A**) Comparison of B cell cluster frequencies from patients at baseline, Time point A and Time point B, stratified by immunotherapy regimen; anti-PD-1 monotherapy and anti-PD-1/LAG-3 combination. Anti-PD-1 monotherapy between baseline and Time point A n=10 and Time point B n=7, anti-PD-1/LAG-3 between baseline and Time point A n=6. (**B**) Comparison of T cell cluster frequencies from patients at baseline, Time point A and Time point B, stratified by immunotherapy regimen; anti-PD-1 monotherapy and anti-PD-1/LAG-3 combination (paired t-test or Wilcoxon depending on normality distribution). Anti-PD-1 monotherapy between baseline and Time point A n=10 and Time point B n=7, and between Time point A and Time point B n=6. Data shown as mean±SEM. *p<0.05; **p<0.01, **p<0.001, ***p<0.0001. Ki-67, proliferation marker-67; LAG-3, lymphocyte activation gene-3; PD-1, programmed cell death protein 1; TGF, transforming growth factor.

In patients receiving anti-PD-1 monotherapy, circulating naïve CD21hi B cells were enriched and class-switched memory IgG1+ cells significantly declined between baseline and Time point A (n=10, paired) (figure 6A). In anti-PD-1/LAG-3, naïve CD21hi B cells showed a trending increase between baseline and Time point A (p=0.0575, n=6, paired), and four mature B cell populations significantly decreased between baseline and Time point A: class-switched memory IgG1+, class-switched memory IgG2+, class-switched memory IgA1+, and non-switched memory B cells (figure 6A). These data indicate that anti-PD-1 monotherapy and anti-PD-1/LAG-3 preferentially promote de novo activation of circulating naïve CD21hi B cells with concomitant contraction of memory pools.

In patients receiving anti-PD-1 monotherapy, four T cell subsets were significantly upregulated on treatment: activated/immunosuppressive CD8+ (TGF-β+VEGF+) T cells increased from baseline to Time point A (n=10, paired), baseline to Time point B (n=7, paired), and Time point A to Time point B (n=6, paired); resting central memory-like CD8+ T cells increased from baseline to Time point A; resting memory CD4+T cells increased from baseline to Time point B and from Time point A to Time point B; and activated/exhausted CD8+ (PD-1+Ki-67+) T cells expanded from baseline to Time point A and baseline to Time point B (figure 6B). No T cell cluster frequency changes were detected in anti-PD-1/LAG-3.

Anti-PD-1 proportionally expands circulating naïve (CD21hi) B cells and effector/proliferative CD8+and memory CD4+ T cells, indicating systemic priming; anti-PD-1/LAG-3 contracts class-switched/unswitched memory B cells and exhibits limited peripheral T cell amplification.

## Discussion

CPI immunotherapy has transformed melanoma treatment, yet the baseline and on-treatment dynamics of how the peripheral adaptive immune response relate to disease activity, toxicity risk and clinical outcomes with CPI, and to different CPI regimens, remain largely unresolved. In this study, we conducted high-dimensional immunophenotyping of circulating B cell alongside T cell subsets in patients with melanoma. Our findings reveal marked differences between patients and healthy individuals, identifying baseline immune signatures associated with disease status, subsequent onset of CPI-related toxicity and survival with CPI and distinct, regimen-specific shifts in circulating B and T cell repertoires during different CPI therapies and combinations.

Alongside an overall proportional decrease of the CD19+ B cell compartment in the peripheral blood of patients compared with healthy volunteers, we found lower expression of B and T cell activation and proinflammatory markers (CD21, CXCR5 on B cells and CD38 and CD27 on T cells); and of IL-2, despite overall low levels in freshly isolated, unstimulated B cells at baseline. Contrastingly, regulatory markers (FoxP3 and TGF-ß on T cells) were increased on circulating immune cells in patients. The patient cohort also features naïve (CD21lo), alternatively-activated (DN, IgD−CD27−) and immunosuppressive (CD95+IL-10+) B cell phenotypes with reduced activation and pro-inflammatory capacity, alongside enriched exhausted (PD-1) and proliferative (Ki67+) T cell populations. These features are in line with enhanced DN2-like, naïve CD21lo B cell and plasmablast frequencies, which have individually been reported across solid tumors such as non-small cell lung cancer, breast cancer and melanoma,^18 37 41 46^ and may point to alteration of a broader systemic regulatory or exhausted response that is skewed towards aberrant extrafollicular differentiation.^47 48^ These features are shared with autoimmune signatures such as in systemic lupus erythematosus (SLE),^49^ whereby naïve B cells are driven into extrafollicular differentiation and expansion into DN2 cells and autoreactive plasmablasts.^50 51^

Our findings also align with the circulating enrichment of exhausted (PD-1+) and activated/proliferating (Ki67+, CD38+HLA-DR+) T cell subsets in patients with melanoma^37^ and breast cancer,^46^ reported by others. Consistent with published reports of immunosuppressive and autoimmune-like features in melanoma,^18 29^ the peripheral immune environment is characterized by impaired effector readiness and enhanced immunoregulatory capacity. In patients with active (metastatic unresected) disease prior to CPI, we found signs of chronic stimulation such as upregulated death receptor CD95 on B cells alongside enrichment of activated Tfh-like cells and DN T cells with proliferating and immunosuppressive phenotypes. Enhanced Tfh-like responses in patients with active disease may reflect a drive towards T cell-mediated stimulation of B cell responses and CD8+ T cell activation in response to chronic stimulation.^52^ Our findings align with elevated, coordinated autoantibody responses in active disease compared with resected disease, identifying features commonly upregulated in cancer and SLE,^29^ and associated with expanded B cells, IL-10^+^ plasmablasts, and exhausted T cell subsets, indicating a coordinated dysregulation of humoral and cellular immunity in active disease. In line with these, our findings thus point to enriched naïve, immunosuppressive, autoimmune-like and chronic inflammatory features of the global circulating adaptive immune compartment in melanoma.

Regulatory aspects of adaptive immunity also associated with reduced risk of toxicity with CPI. Higher baseline frequencies of activated DN T cells with regulatory features (VEGF+TGF-ß+) in patients who did not subsequently develop irAEs suggest that aspects of peripheral immune tolerance may provide protective mechanisms against toxicity.^18^ Baseline PD-1 expression was increased on T cells of patients that subsequently developed irAEs, perhaps reflecting enhanced capacity for re-stimulation during CPI in these patients, and therefore these signatures could identify a priori patients more prone to irAE development. Together, these observations underscore the interplay between protective tolerance mechanisms and antitumor immune activation, aligning with reports that irAE onset can coincide with enhanced CPI responses and survival.^53 54^

On the other hand, different adaptive immune features may link with CPI treatment responses. Previous studies have reported enriched TGF-ß+ B cells in melanoma^20^ and VEGF+ B cells in cancer and chronic inflammation.^28^ Here we show that increased frequency of several regulatory/immunosuppressive B and T cell subsets prior to treatment highlight disease progression and survival after CPI. Plasmablasts, transitional B cells, FoxP3+ T cells and resting central memory-like CD8 T cells at baseline predict worse OS, and high levels of naïve CD21lo B cells, PD-1-expressing CD8+ T cells and CD4+ Tfh like T cells correlate with reduced EFS. Combined, these suggest that patients with an underdeveloped/immature or an immunosuppressive and exhausted adaptive immune compartment may have reduced capacity for cytotoxic activation and stimulation of effective antitumor responses, linked with less favorable outcomes with subsequent CPI.^18 48 55 56^ Contrastingly, higher adaptive immune response fitness, characterized by enrichment of resting effector memory CD4+ T cells at baseline, however, predicts more favorable outcomes, likely denoting a well-regulated T cell compartment in these patients with long-term immunity primed for boosting cytotoxic T cells.^57 58^

We furthermore found evidence of dynamic B and T cell shifts during CPI. Elevated abundance of the whole B cell, activated (CD21hiCXCR5+HLA-DR+CD40+) naïve B cell and rapidly-responding plasmablast frequencies during CPI therapy coincide with a proportional decline in class-switched memory and non-switched memory B cell subsets. Alongside, several activated CD8+ T cell subsets with exhausted (PD-1) and immunosuppressive features (TGF-ß+VEGF+) and central memory-like CD4+ T cells proportionally expanded, while resting effector memory CD4+ T cells declined on treatment, perhaps reflecting a drive towards activated effector T cell signatures in response to CPI. Furthermore, while phenotypically-exhausted and immunosuppressive cytotoxic T cell populations increased during CPI, expression of TGF-ß declined on-treatment, reflecting CPI-induced reinvigoration of T cell pro-inflammatory properties. These dynamic shifts favor activated B and Tcell subsets, indicating a reversal of some immune exhaustion, and suggest de novo activation of naïve B cells during CPI occurring concurrently with broad T cell stimulation. These findings also mark the importance of B cells in effective immunotherapy responses: de novo activation of the B cell compartment likely favoring renewed antigen or neoantigen stimulation and re-initiation of antigen presentation may revive adaptive B and T cell responses and better tumor control. Our data is in concordance with recent findings of proportionally expanded circulating B cells and plasmablasts, alongside increased peripheral frequencies of activated Ki67+ effector T cells during CPI therapy.^18 34 38^

Significant changes in the frequency of several B and T cell subsets during the first 6 weeks of CPI treatment predict improved survival outcomes. An increase in class-switched memory (IgG2+) B cells during therapy is associated with improved EFS. However, patients that favor naïve CD21lo or double negative (DN2, PD-1+DN2) B cells during treatment correlate with reduced OS and EFS. This suggests that an expansion of immature and alternatively-activated B cell responses during CPI confers worse prognosis, in line with reports of atypical B cell features, namely CD21-CD27-IgD- B cells on treatment correlate with worse CPI outcomes in non-small cell lung cancer.^59^ In circulating T cells, we observe that coordinated expansion of a diverse range of T cell populations correlate with better outcomes, suggesting a globally engaged and regulated T cell response to immunotherapy.

Immunotherapy responses differ significantly between patients and can also depend on the type or combination of CPI.^15 60 61^ Proportional expansion of activated naïve B cells, activated CD8 T cells and CD4 memory T cells in the circulation of patients on treatment with anti-PD-1 monotherapy may denote initiation of adaptive immunity.^9 11 12 18 34 62^ Anti-PD-1 monotherapy relieves exhaustion of pre-existing antigen-experienced lymphocytes without simultaneously altering early priming or germinal center (GC) regulation, thereby promoting adaptive B cell and T cell expansion. It is also possible that LAG-3 blockade may restrain GC or memory B cell maintenance through dampened Tfh-mediated GC support. The absence of peripheral effector T cell amplification with anti-LAG-3 combination therapy may indicate early tissue-resident activation or greater intratumoral recruitment, reducing their proportional representation in blood.^20 29^ These may point to circulating B and T cell responses, with anti-PD-1 monotherapy triggering effective stimulation of the systemic immune response.

Several limitations should be considered when interpreting our findings. Patients with more advanced and aggressive disease are more likely to receive combination CPI regimens than monotherapy, which may introduce some bias when studying adaptive immune responses in each treatment group. Furthermore, our mass cytometry panel was primarily designed to achieve high-resolution profiling of B cell populations, with expansion to capture major T cell subsets. Consequently, certain T cell populations could not be fully resolved. For example, the absence of CD45RA and CCR7 limits the distinction of naïve T cells, which are likely included within resting central memory-like clusters, and regulatory T cells are likely included within the mixed FoxP3+ population. Our analyses were based on proportional rather than absolute cell frequencies; therefore, observed alterations in specific subsets may, in part, reflect relative expansion of other populations rather than true numerical contraction. Our conclusions are, however, supported by concordant changes across multiple-related immune subsets rather than isolated shifts in single populations. Future studies may incorporate larger, balanced cohorts, expanded marker panels, and absolute cell quantification to help refine subset definition.

In conclusion, our findings reveal dynamic circulating B cell and T cell states aligned with clinical trajectories in CPI therapy. Baseline enrichment of likely extrafollicular B cell populations (naïve CD21lo, DN, plasmablasts) and resting CD8/exhausted T cells, indicates reduced effector readiness and is associated with poorer outcomes, whereas resting CD4+ effector memory T cells denote favorable, recall-competent adaptive immunity. We further identify PD-1^+^ T cells as pretreatment predictors of toxicity and show that features of persistent activation (CD95+ B cells; Tfh/DN T cells) mark active disease. Importantly, a subset of recurrent immune features may be prioritized for further study and validation, including extrafollicular B cell (naïve CD21lo, transitional, DN, plasmablast) subsets and exhausted/regulatory T cell (PD-1+ CD8+, Tfh CD4+, FoxP3+, DN TGFß+VEGF+) states, which were associated with adverse outcomes at baseline, and across longitudinal and clinical endpoint analyses for the B cell signatures. In contrast, expansion of activated/proliferating T cells and class-switched B cells during treatment suggests effective adaptive immunity and predicts improved outcomes. Together these features represent dynamic indicators of clinical outcomes and provide a translational framework, positioning coexisting peripheral Bcell maturation alongside global T cell expansion as candidate biomarkers to optimize CPI in melanoma. Our findings support integrated pretreatment and on-treatment humoral and cellular adaptive immune monitoring to inform response, toxicity risk, and clinical management decisions.

## Supplementary material

10.1136/jitc-2026-015585 online supplemental file 1

## Data Availability

Data are available upon reasonable request.
