# Supplementary material for: Circulating B cell and T cell activation states predict clinical outcomes in melanoma and reveal dynamic immune reinvigoration with checkpoint inhibitor immunotherapy
Source: J Immunother Cancer. 2026 Aug 7;14(8):e015585. doi: 10.1136/jitc-2026-015585 (PMC13475463; doi:10.1136/jitc-2026-015585)
Supplement: Supplementary file 1 [file jitc-14-8-s001.docx]

**Booth et al., Supplementary Material**

**Supplementary Figures**

**Supplementary Figure 1. Flowchart schematic of healthy volunteer and melanoma patient sample cohort with longitudinal samples on treatment with CPI.** Peripheral blood sampling protocol from healthy volunteers and patients with stage II/III/IV melanoma planned to receive checkpoint inhibitor immunotherapy were recruited and baseline peripheral blood sampling was performed. Subsequent on-treatment samples were taken at week 6-8 for Timepoint A and week 12 for Timepoint B. Flowchart describes the number of patients included at each sampling timepoint, the checkpoint inhibitor the patients received, and details of attrition (samples lost either due to clinical access restriction, discontinued treatment or disease progression).

**Supplementary Figure 2. Gating Strategy for mass cytometry analyses, where events are sequentially gated using FlowJo to obtain purified B and T cell populations from stained PBMCs.**

**CD45+**

**Supplementary Figure 3. Naïve, immunosuppressive and alternatively activated circulating humoral immune phenotypes enriched in peripheral blood of melanoma patients (n=24) compared to healthy volunteers (n=24).** (A) Cluster heatmap showing median scaled expression of the 20 unsupervised metaclusters, and the 14 merged B cell subsets for the melanoma patient vs healthy volunteer cohort. (B) Table detailing how the unsupervised clusters were merged into the defined B cell subsets. (C) Differential abundance (DA) test showing significantly differentially expressed unsupervised B cell clusters between HVs and melanoma patients. Normalised frequency indicates the relative abundance of each cluster per sample, and logFC indicates the change in frequency of each cluster between the two cohorts. Green bars indicate statistically significant differential expression after multiple test correction (BH-adjusted p values). (D) Relative abundance of B cell clusters as a proportion of CD19+ cells in healthy volunteers and melanoma patients (divided by stage: melanoma (all), n=24; stage 2, n=3; stage 3, n=11; stage 4, n=10), HV (N=25), (One-way ANOVA with Dunnett’s multiple comparisons test).

**Supplementary Figure 4. Proportion of peripheral blood T cells are unchanged between healthy volunteers and melanoma patients.** (A) Relative abundance of total T cells (CD3+) as a proportion of CD45+ cells in healthy volunteers and melanoma patients (divided by stage: melanoma (all), n=24; stage 2, n=3; stage 3, n=11; stage 4, n=10) (one-way ANOVA with Tukey’s multiple comparisons test). B) Spearman rank correlation plots showing percentage cell frequencies vs age of melanoma patients (n=24) for the significantly differentially expressed B and T cell clusters between HV and melanoma (Spearman rank correlation).

**Supplementary Figure 5. Visualising B and T cell clusters from the pre-treatment patient cohort (N=24).** (A) Cluster heatmap of 14 merged B cell clusters. (B) t-SNE of 14 B cell subsets. (C) Cluster heatmap of 8 T cell clusters. (D) t-SNE of 8 T cell subsets. (C) Multivariate cox proportional hazard model that uses pre-treatment B and T cell frequencies to predict overall and event-free survival (n=24).

**Supplementary Figure 6. Peripheral blood phenotyping of B cells from longitudinal samples of baseline and on treatment with checkpoint inhibitors.** (A) Cluster heatmap showing median scaled expression of the 20 unsupervised metaclusters, and the 14 merged B cell subsets for the melanoma patient pre and post treatment cohort. (B) Table detailing the how the unsupervised clusters were merged into the defined B cell subsets. (C) Abundance bar chart representing proportions of merged clusters per donor between pre-treatment and on-treatment melanoma patients. (D) Multivariate cox proportional hazard model for assessing changes in B cell subsets on treatment and association with prognosis (n=18). (E) Kaplan-Meier analysis for on-treatment B cell frequencies and their association with overall and event-free survival (n=18) (Logrank Mantel-Cox test).

**Supplementary Figure 7.** **Peripheral blood phenotyping of T cells from longitudinal samples of baseline and on treatment with checkpoint inhibitors.** (A) Abundance bar chart representing proportions of T cell clusters per donor between pre-treatment and on-treatment melanoma. (B) DA analysis comparing unpaired samples of baseline (N=24) and on-treatment samples (N=36). (C) Relative abundance of T cell subsets that are significantly differentially expressed in an unpaired analysis of samples pre-treatment and on-treatment (diffcyt DA test). (D) Multivariate cox proportional hazard model for assessing changing changes in T cell subsets on treatment and association with prognosis. (E) Kaplan-Meier analysis for on-treatment T cell frequencies and their association with overall and event-free survival (n=18) (Logrank Mantel-Cox test).

**Supplementary Tables**

**Supplementary Table 1.** Summary of melanoma patient characteristics, treatment and response details. Patients in this cohort did not receive neoadjuvant therapy.

| Patient ID | Stage | Previous Medical History | Previous systemic treatment for melanoma | Disease at baseline (AD/RD) | Checkpoint Inhibitor Received | Toxicity Grade |
| --- | --- | --- | --- | --- | --- | --- |
| M1 | 3 | Dermatosis - Grover's disease | No | AD | Anti-PD1 (Pembrolizumab) | 1 |
| M2 | 3 | Psoriasis; Diabetes 2; HTN; CABG | (Dabrafenib/Trametinib) | AD | Anti-PD1/anti-LAG3  (Nivolumab/ Relatlimab) | 0 |
| M3 | 3 | None | No | RD | Anti-PD1  (Nivolumab) | 0 |
| M4 | 4 | None | No | AD | Anti-PD1  (Pembrolizumab) | 1 |
| M5 | 4 | None | No | AD | Anti-PD1  (Pembrolizumab) | 0 |
| M6 | 3 | None | (Dabrafenib/Trametinib) | AD | Anti-PD1/anti-LAG3  (Nivolumab/ Relatlimab) | 0 |
| M7 | 3 | None | No | AD | Anti-PD1/anti-LAG3  (Nivolumab/ Relatlimab) | 2 |
| M8 | 4 | Diabetes | (Dabrafenib/Trametinib) | AD | Anti-PD1/anti-LAG3  (Nivolumab/ Relatlimab) | 0 |
| M9 | 4 | None | (Dabrafenib/Trametinib) | AD | Anti-PD1/anti-CTLA4  (Ipililumab/ Nivolumab) | 3 |
| M10 | 3 | None | No | RD | Anti-PD1  (Pembrolizumab) | 1 |
| M11 | 2 | None | No | RD | Anti-PD1  (Pembrolizumab) | 2 |
| M12 | 4 | None | No | AD | Anti-PD1  (Pembrolizumab) | 0 |
| M13 | 4 | None | No | AD | Anti-PD1/anti-CTLA4  (Ipililumab/ Nivolumab) | 0 |
| M14 | 2 | None | No | RD | Anti-PD1  (Pembrolizumab) | 1 |
| M15 | 3 | Prostate cancer | No | RD | Anti-PD1  (Pembrolizumab) | 0 |
| M16 | 4 | None | (Dabrafenib/Trametinib) | AD | Anti-PD1/anti-LAG3  (Nivolumab/ Relatlimab) | 0 |
| M17 | 4 | None | No | AD | Anti-PD1/anti-CTLA4  (Ipililumab/ Nivolumab) | 1/2 |
| M18 | 2 | Hepatic steatosis | No | RD | Anti-PD1 (Pembrolizumab) | 0 |
| M19 | 4 | None | No | AD | Anti-PD1/anti-CTLA4  (Ipililumab/ Nivolumab) | 3 |
| M20 | 3 | BCC; TIA; HTN; anaemia; DVT | No | RD | Anti-PD1 (Pembrolizumab) | 1 |
| M21 | 3 | None | No | AD | Anti-PD1 (Pembrolizumab) | 4 |
| M22 | 3 | Polymyalgia rheumatica; | No | AD | Anti-PD1 (Pembrolizumab) | 3/4 |
| M23 | 3 | None | No | AD | Anti-PD1 (Pembrolizumab) | 1/2 |
| M24 | 4 | Transverse myelitis; hypothyroid | No | AD | Anti-PD1/anti-LAG3  (Nivolumab/ Relatlimab) | 0 |

Acronyms: HTN hypertension, CABG coronary artery bypass graft, BCC basal cell carcinoma, TIA transient ischaemic attack, DVT deep vein thrombosis, AD active disease, RD resected disease

**Supplementary Table 2**. Summary of cohort age and gender between baseline melanoma patients and healthy volunteers

| Cohort | Number of samples | Median Age | Standard Deviation (SD) of Age | Gender Split |
| --- | --- | --- | --- | --- |
| Healthy Volunteers | 24 | 57.5 | 16.73 | 44% Male 56% Female |
| Pre-treatment Melanoma | 25 | 72.5 | 10.71 | 66% Male 33% Female |

**Supplementary Table 3.** Full CyTOF Panel of markers for phenotyping of circulating B cells (CD19+) and T cells (CD3+) from PBMCs

| Metal Label | Antigen | Used for clustering (Y/N) | Manufacturer | Clone | Titre |
| --- | --- | --- | --- | --- | --- |
| Y89Di | CD45 | No | Standard Biotools | HI30 | 1:167 |
| Rh103Di | Live_Dead | No | Standard Biotools | N/A | 1:500 |
| Cd106Di | CD19 | No | Biolegend | HIB19 | 1:200 |
| Cd110Di | IgA1 | Yes | Southern Biotech | B3506B4 | 1:100 |
| Cd112Di | IgE | Yes | Biolegend | MHE-18 | 1:100 |
| Cd113Di | IgG3 | Yes | BD Biosciences | HP6050 | 1:100 |
| Cd114Di | TNFa | Yes | Standard Biotools | Mab11 | 1:400 |
| CD116DI | IFNg | Yes | Standard Biotools | B27 | 1:200 |
| Ce140Di | EQ_Beads | No | N/A | N/A | N/A |
| Pr141Di | CD3 | No | Standard Biotools | UCHT1 | 1:100 |
| Nd142Di | IL4 | Yes | Standard Biotools | MP4-25D2 | 1:400 |
| Nd143Di | IgG1 | Yes | Miltenyi Biotech | IS11-12E4.23.20 | 1:100 |
| Nd144Di | CD38 | Yes | Standard Biotools | HIT2 | 1:200 |
| Nd145Di | IgG2 | Yes | Sourthern Biotech | HP6014 | 1:100 |
| Nd146Di | IgD | Yes | Standard Biotools | IA6-2 | 1:200 |
| Sm147Di | VEGF | Yes | R&D systems | VG1 | 1:200 |
| Nd148Di | IL2 | Yes | Biolegend | MQ1-17H12 | 1:400 |
| Sm149Di | CD25 | Yes | Standard Biotools | 2A3 | 1:143 |
| Nd150Di | CD138 | Yes | Standard Biotools | DL101 | 1:200 |
| Eu151Di | HLA-DR | Yes | Standard Biotools | G46-6 | 1:400 |
| Sm152Di | CD21 | Yes | Standard Biotools | BL13 | 1:200 |
| Eu153Di | Ig_Lambda | No | Standard Biotools | MHL-38 | 1:200 |
| Sm154Di | FoxP3 | Yes | Biolegend | 259D | 1:200 |
| Gd155Di | PD-1 | Yes | Standard Biotools | EH12.2H7 | 1:100 |
| Gd156Di | PD-L1 | Yes | Standard Biotools | 29E.2A3 | 1:100 |
| Gd158Di | CD10 | Yes | Standard Biotools | HI10a | 1:143 |
| Tb159Di | CD22 | Yes | Standard Biotools | HIB22 | 1:200 |
| Gd160Di | Ig_Kappa | No | Standard Biotools | MHK-49 | 1:200 |
| Dy161Di | CD5 | Yes | Biolegend | UCHT2 | 1:100 |
| Dy162Di | CD79B | Yes | Standard Biotools | CB3-1 | 1:200 |
| Dy163Di | BCL-6 | Yes | Standard Biotools | K112-91 | 1:143 |
| Dy164Di | CD95 | Yes | Standard Biotools | DX2 | 1:200 |
| Ho165Di | CD40 | Yes | Standard Biotools | 5C3 | 1:200 |
| Er166Di | IL-10 | Yes | Standard Biotools | JES3-9D7 | 1:100 |
| Er167Di | CD27 | Yes | Standard Biotools | L128 | 1:250 |
| Er168Di | Ki-67 | Yes | Standard Biotools | B56 | 1:125 |
| Tm169Di | CD24 | Yes | Standard Biotools | ML5 | 1:200 |
| Er170Di | TGF-Beta | Yes | Biolegend | S20006A | 1:100 |
| Yb171Di | CXCR5 | Yes | Standard Biotools | RF8B2 | 1:133 |
| Yb172Di | IgM | Yes | Standard Biotools | MHM-88 | 1:333 |
| Yb174Di | IgG4 | Yes | Invitrogen | HP6023 | 1:200 |
| Lu175Di | CD28 | Yes | Biolegend | CD28.2 | 1:200 |
| Yb176Di | CD127 | Yes | Standard Biotools | A019D5 | 1:400 |
| Ir191Di | DNA_01 | No | Standard Biotools | N/A | 1:300 |
| Ir193Di | DNA_02 | No | Standard Biotools | N/A | 1:300 |
| Pt195Di | CD20 | No | Biolegend | 2H7 | 1:100 |
| Pt196Di | CD8a | Yes | Biolegend | RPA-T8 | 1:400 |
| Pt198Di | CD4 | Yes | Biolegend | RPA-T4 | 1:200 |
| Bi209Di | CD16 | No | Standard Biotools | 3G8 | 1:400 |

**Supplementary Table 4.** Summary of melanoma patient information used for survival analysis of overall and event-free survival.

| Patient ID | Start of Treatment | Date of death or last follow up | Death (Y/N) | Days for overall survival | Date of progression or last follow up* | Event (Y/N) | Days for event-free survival |
| --- | --- | --- | --- | --- | --- | --- | --- |
| M1 | 22/03/2018 | 03/05/2024 | no | 2234 | 03/05/2024 | no | 2234 |
| M2 | 09/08/2024 | 03/11/2025 | no | 451 | 28/10/2024 | yes | 80 |
| M3 | 23/01/2019 | 17/07/2025 | no | 2367 | 17/07/2025 | no | 2367 |
| M4 | 03/01/2020 | 04/11/2025 | no | 2132 | 04/11/2025 | yes | 2132 |
| M5 | 20/05/2020 | 20/11/2025 | no | 2010 | 20/10/2020 | yes | 153 |
| M6 | 26/09/2024 | 24/11/2025 | no | 424 | 14/02/2025 | yes | 141 |
| M7 | 18/06/2024 | 21/11/2025 | no | 521 | 24/03/2025 | yes | 279 |
| M8 | 19/08/2024 | 29/01/2025 | yes | 163 | 10/12/2024 | yes | 113 |
| M9 | 18/03/2024 | 06/11/2025 | no | 598 | 29/11/2024 | yes | 256 |
| M10 | 30/11/2022 | 30/10/2025 | no | 1065 | N/A | no | 1065 |
| M11 | 01/02/2023 | 25/09/2025 | no | 967 | N/A | no | 967 |
| M12 | 25/05/2023 | 26/08/2024 | yes | 459 | 30/11/2023 | yes | 189 |
| M13 | 11/07/2023 | 16/10/2025 | no | 828 | 16/10/2025 | no | 828 |
| M14 | 25/07/2023 | 25/09/2025 | no | 793 | N/A | no | 793 |
| M15 | 30/08/2023 | 31/07/2025 | no | 701 | N/A | no | 701 |
| M16 | 20/09/2024 | 21/03/2025 | yes | 182 | 05/11/2024 | yes | 46 |
| M17 | 17/10/2023 | 09/10/2025 | no | 723 | 09/10/2025 | no | 723 |
| M18 | 04/12/2023 | 23/10/2025 | no | 689 | N/A | no | 689 |
| M19 | 23/01/2024 | 20/11/2025 | no | 667 | 09/10/2025 | yes | 625 |
| M20 | 04/03/2024 | 13/11/2025 | no | 619 | N/A | no | 619 |
| M21 | 26/02/2024 | 21/04/2025 | yes | 420 | 21/04/2025 | yes | 420 |
| M22 | 04/03/2024 | 16/10/2025 | no | 591 | 16/10/2025 | no | 591 |
| M23 | 30/04/2024 | 20/11/2025 | no | 569 | 20/11/2025 | no | 569 |
| M24 | 05/09/2024 | 22/11/2025 | no | 443 | 21/05/2025 | yes | 258 |

*For adjuvant patients with resected disease at baseline (no measurable disease), date of death or last follow up was used for event-free survival instead of progression as endpoint.

**Supplementary Materials and Methods**

*Peripheral blood mononuclear cell isolation and cryopreservation*

Peripheral blood mononuclear cell (PBMC) processing and cryopreservation was conducted according to previously established protocols. Whole blood was collected in 10ml purple-capped 10ml EDTA-coated tubes and were kept on a roller for a maximum of 4 hours before processing. Phosphate buffered saline (PBS) was used to dilute the whole blood 1:1 in 50ml Falcon™ tubes, followed by careful layering into a 50ml Leucocep tube containing 15ml Ficoll-Paque™ Plus. Leucocep tubes were centrifuged at 1020g for 15 minutes at room temperature (RT) (acceleration 3, deceleration 0), and the PBMC layer was harvested and subsequently washed with PBS at 1020g for 10 minutes. Cells were washed once with 5ml Red Cell Lysis Buffer at 370g for 5 minutes, and again with 5ml RPMI at 370g for 5 mins. Isolated PBMCs were immediately resuspended in Freezing Solution A and B (Foetal Bovine Serum (FBS), RPMI1640 and Dimethyl Sulfoxide (DMSO at a 4:4:1 ratio) and stored in a -80°C freezer in a Mr Frosty, with subsequent transfer to liquid nitrogen until time of assay.

*Thawing of PBMCs for mass cytometry*

Cryopreserved aliquots of PBMCs were removed from liquid nitrogen and thawed in a 37°C water bath when required for CyTOF staining. Thawed cells were added to 10ml warmed RPMI1640 cell culture medium supplemented with FBS and P/S and centrifuged at 300g for 10 minutes. Cells were rested in the incubator at 37°C for 1 hour and subsequently washed in RPMI as above. PBMCs were counted and viability was tested using Denovix Cell Drop cell counter with AO/PI dye at a 1:1 dilution.

*Mass Cytometry cell staining and data acquisition*

Approximately 3-5 million PBMCs per sample were used for CyTOF staining. PBMCs were added to blue filter-capped FACS tubes (Fisher Scientific) and washed with 3ml of MaxPar Cell Staining Buffer (Standard Biotools) at 800g for 5 minutes. Cell pellets were resuspended in approximately 80ul buffer and 5ul of Fc Blocking Solution (Human Trustain Fc Blocking Solution (Standard Bio)) was added to the suspension for 10 minutes at room temperature. Metal-tagged antibodies were micro-centrifuged at 10,000g for 5 minutes to prevent antibody aggregates, and the extracellular antibody master mix was added to the cells, followed by a gentle vortex and incubation for 30 minutes. Cells were washed with 1ml MaxPar Cell Staining Buffer, and 1uM Rhodium (Rh103) (used as a live/dead cell stain), was added to each sample for 15 minutes at RT. Cells were washed as before, and fixed using 1ml MaxPar Fix I Buffer (Standard Bio) for 15 minutes at RT. This was followed by two washes in MaxPar S Permeabilisation Buffer (Standard Bio), and the intracellular antibody master mix was added to each sample and incubated for 30 minutes at room temperature. After another wash in MaxPar Cell Staining Buffer, cells were incubated overnight at 4°C in intercalation solution containing Iridium DNA Intercalator (Standard Bio) and MaxPar Fix and Perm Buffer (Standard Bio) at a 1:350 concentration. Finally, prior to acquisition on a Helios™ Mass Cytometer, cells were washed with MaxPar Cell Staining Buffer (Standard Bio) and twice in Milli-Q water. Data was acquired at a rate of 300-400 events per second. Each batch of samples included a standardised healthy control PBMC sample along with the patient samples for quality control and consistency, as well as a standardised cone PBMC sample with every batch.

*Mass cytometry and cluster analysis using the FlowSOM algorithm*

FCS files obtained from the Helios™ were normalised and concatenated using the Fluidigm CyTOF software. Files were uploaded to BD FlowJo software for data clean up and gating of live, intact, singlet cells using EQ beads, Iridium intercalator for DNA staining, Rhodium for live/dead staining and event length. Purified B cell and T cell populations were gated following the gating strategy in Supplementary Figure S1.

CD19+ and CD3+ fcs. files were analysed downstream using R packages CATALYST, diffCYT, FlowSOM, edgeR and flowCORE, all of which can be found on the Bioconductor terminal. B and T cell phenotypes were determined using the unsupervised FlowSOM clustering algorithm (Van Gassen et al., 2015), and distinct metaclusters were defined according to median scaled expression of 34 markers for B cells and 22 markers for T cells, as previously described (Nowicka et al., 2017, Weber et al., 2018). Data were then visualised using t-SNE or UMAP algorithm for dimensionality reduction.

*Statistical analyses*

Spearman Rank Correlations: To assess the relationship between circulating B and T cell subsets we calculated pairwise spearman rank correlations between all B cell and T cell clusters across patients. For each B-T cell cluster pair, the correlation coefficient and corresponding p value were generated using cor.test, and p-values were adjusted using the Benjamini-Hochberg false discovery rate (FDR) method. To facilitate the association between B/T cell cluster frequency and survival, patients were stratified into high (>75^th^ percentile), intermediate or low (<25^th^ percentile) frequency groups for each cluster pairing. Survival analysis for these cluster combinations was carried out using the high vs low designations as the cut offs, and Kaplan Meier plots were generated with log-rank test for statistical analysis.

**Supplementary References**

Van Gassen, S., et al., *FlowSOM: Using self-organizing maps for visualization and interpretation of cytometry data.* Cytometry A, 2015. **87**(7): p. 636-45

Weber, L.M., et al., *diffcyt: Differential discovery in high-dimensional cytometry via high-resolution clustering.* Commun Biol, 2019. **2**: p. 183.

Nowicka M, et al., *CyTOF workflow: differential discovery in high-throughput high-dimensional cytometry datasets*. F1000Res. 2017. 26;6:748.
